# PCA2GO: a new multivariate statistics based method to identify highly expressed GO-Terms

**DOI:** 10.1186/1471-2105-11-336

**Published:** 2010-06-21

**Authors:** Marc Bruckskotten, Mario Looso, Franz Cemiĉ, Anne Konzer, Jürgen Hemberger, Marcus Krüger, Thomas Braun

**Affiliations:** 1 Department of Cardiac Development and Remodelling, Max-Planck-Institute for Heart and Lung Research, Bad Nauheim, Germany; 2 Institute for Biochemical Engineering and Analytics, University of Applied Sciences Giessen-Friedberg, 35390 Giessen, Germany

## Abstract

**Background:**

Several tools have been developed to explore and search Gene Ontology (GO) databases allowing efficient GO enrichment analysis and GO tree visualization. Nevertheless, identification of highly specific GO-terms in complex data sets is relatively complicated and the display of GO term assignments and GO enrichment analysis by simple tables or pie charts is not optimal. Valuable information such as the hierarchical position of a single GO term within the GO tree (topological ordering), or enrichment within a complex set of biological experiments is not displayed. Pie charts based on GO tree levels are, themselves, one-dimensional graphs, which cannot properly or efficiently represent the hierarchical specificity for the biological system being studied.

**Results:**

Here we present a new method, which we name PCA2GO, capable of GO analysis using complex multidimensional experimental settings. We employed principal component analysis (PCA) and developed a new score, which takes into account the relative frequency of certain GO terms and their specificity (hierarchical position) within the GO graph. We evaluated the correlation between our representation score *R *and a standard measure of enrichment, namely *p*-values to convey the versatility of our approach to other methods and point out differences between our method and commonly used enrichment analyses. Although *p *values and the *R *score formally measure different quantities they should be correlated, because relative frequencies of GO terms occurrences within a dataset are an indirect measure of protein numbers related to this term. Therefore they are also related to enrichment. We showed that our score enables us to identify more specific GO-terms i.e. those positioned further down the GO-graph than other common tools used for this purpose. PCA2GO allows visualization and detection of multidimensional dependencies both within the acyclic graph (GO tree) and the experimental settings. Our method is intended for the analysis of several experimental sets, not for one set, like standard enrichment tools. To demonstrate the usefulness of our approach we performed a PCA2GO analysis of a fractionated cardiomyocyte protein dataset, which was identified by enhanced liquid chromatography-mass spectrometry (GeLC-MS). The analysis enabled us to detect distinct groups of proteins, which accurately reflect properties of biochemical cell fractions.

**Conclusions:**

We conclude that PCA2GO is an alternative efficient GO analysis tool with unique features for detection and visualization of multidimensional dependencies within the dataset under study. PCA2GO reveals strongly correlated GO terms within the experimental setting (in this case different fractions) by PCA group formation and improves detection of more specific GO terms within experiment dependent GO term groups than standard *p *value calculations.

## Background

The advent of high-throughput techniques, which allow rapid acquisition of vast data sets, has created the need for fast and reliable functional annotation of molecules. A typical DNA microarray [1] or high throughput LC- mass-spectrometry experiment [2] often leads to the identification of changes in the expression of hundreds if not thousands of molecules. The identification of pathways or biological processes that are affected in a given experimental set-up requires functional annotation of multiple gene products, which can be achieved by association with a set of annotation terms. The Gene Ontology project is a collaborative effort to provide a controlled vocabulary to describe gene product attributes in different organisms [3]. The use of Gene Ontology Annotations (GOA) in high-throughput contexts has become a widespread practice to gain insight into the potential biological meaning of profiling experiments. GO provides structured, controlled vocabularies and classifications such as *biological process*, *molecular function *and *cellular component*. The relationship between GO terms can be described by a directed acyclic graph (DAG). Relations within GO terms, like *is_a *and *part_of *or *regulates *are represented by direct edges. The true-path rule postulates that an annotated gene is also associated with less specific parents of that term. Explicitly, *the pathway from a child term all the way up to its top-level parent(s) must always be true *[3]. Publicly available software packages like DAVID [4], GOstat [5], FatiGO [6], GOrilla [7], BINGO [8], blast2GO [9] and others use various approaches to visualize, filter and search the GO database. Web-based applications allow users to download their findings in graphic formats or as text tables. One of the most common GO applications is to test datasets for gene enrichment. Enrichment of single GO terms might reveal potential functional characteristics of a given dataset. Typically, enrichment analyses are based on hyper geometric or binomial models. Most software tools use similar algorithms although the accepted input data might differ. Some tools need a target as well as a background set of genes as input, while others use only a default background set. A more general discussion of the advantages of different approaches can be found in [10]. Here, we investigated the usability of our new GO-based method applied to modern high-throughput techniques, like mass spectrometry using the SILAC approach. Naturally, our method has to be adapted to the question addressed by the experiment. In this case the purity of certain pre-sorted cell fraction derived from LC-MS needed to be verified. We showed that our methods enabled us to check whether it is suitable to verify the purity of a certain cell fraction using the GOA platform. Furthermore, we were able to show, that our *R *score combined with principal component analysis (PCA) is capable to detect strongly represented branches of the GO DAG (i.e. gene products included within these branches) comparable to the output of standard *p *values enrichment tools.

## Results

To detect strongly represented GO branches more specifically than with commonly used methods, we developed a specific representation score *R *that combined with PCA takes into account relative frequencies of gene product occurrences within the data set and topological ordering of the GO-DAG. To reveal specific dependencies within the dataset, we performed a PCA on that new score *R*. In order to demonstrate the performance of our method, we applied it to an experimental dataset derived from fractionated cardiomyocyte proteins, which was obtained by enhanced liquid chromatography-mass spectrometry (GeLC-MS). Our analysis enabled us to accurately detect distinct groups of proteins that are localized in specific cell fractions.

### Data processing and R score assignment

In a first step we associated each protein to cytosolic, membrane, nuclear or non-specific fraction as described in the methods section below. We identified 179 proteins, which were associated with the cytosolic-, 328 with the membrane-, and 45 with the nucleus-fraction of cardiomyocytes. An additional group of 1284 cardiomyocyte proteins was detected but placed in the "non-specific" fraction, since proteins from this group were found in significant amounts in more than one fraction. This non-specific fraction represented an experimental internal standard as detailed in the methods section. GO terms were individually assigned to our protein lists, based on the IPI database from EBI. Since we were interested in the quality and efficiency of our fractionization, we used only GO terms that belong to the classification "*cellular compartment"*. As a next step, we evaluated a *Score of Representation (R) *for each GO term as detailed later in the text. These *R *scores were then used for PCA to unravel multivariate dependencies inherent in our data. Our *R *score mapped the GO terms on the interval [0,1]. Furthermore the *R *score provided information about the order of the GO terms. Within these groups of GO terms, the *R *score of a more specific term was lower than that of a less specific one, even if the relative frequency of the gene products represent by the GO term was almost equivalent. This was due to our definition of *R*. When the GO terms were sorted by decreasing *R score values*, more specific terms were found at the end of the list.

### PCA results

**Figure 1:**
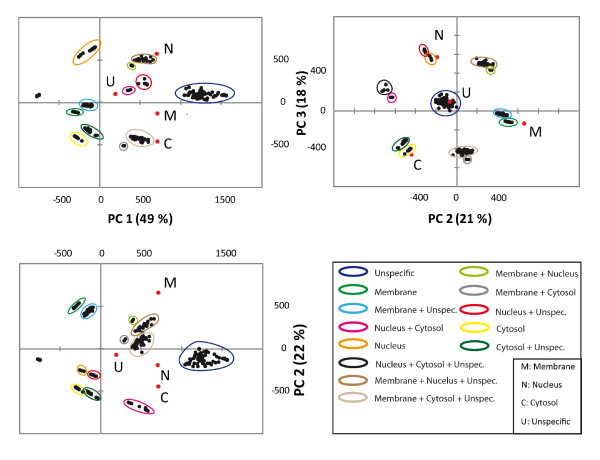
**Bi-plot of fraction data**. The first Principal Component was correlated with distinct PC variables (covariance matrix **S**). Therefore, the hierarchy becomes visible in plots of GO-terms of datasets, which were not highly affected by a characteristic value of the PC variable. The group of unspecific terms was strongly correlated with PC1 (*Loadings *are marked in red). The correlation of the PCA variable with a PC is visible and allows identification of PCA, which are connected to the PCA variable. Identified groups, which were connected to a PCA variable in the bi-plot, were coloured according to their subcellular localization.

The output of a PCA yielded in a series of graphs, namely so called bi-plots (as shown in Figure 1). The bi-plot is a scatter plot that graphically displays a matrix of rank two, formed by the observations (rows) and the variables (columns). In our case, the observations represented our fractions and the variables the GO terms belonging to each fraction. The correlations of the different fractions among the principal components are listed in Table 1. As indicated in the table, the fraction of membrane GO terms are strongly correlated with PC2, the fraction of nucleus GO terms are strongly correlated with PC3 and GO terms belonging to the fraction of non-specific GO terms (prior to PCA) are strongly correlated to PC4. PC scores are the coordinates of our observables (*cytosolic, membrane, nucleus *or *non-specific*) in a subspace spanned by the loading vectors. The mathematical formalism of PCA can be found in the methods section. The first PC is always related to the number of predecessors to a GO term, since this number exhibits the highest variance in the dataset under study. Naturally, due to several branches of the GO hierarchy the exact number cannot be measured exactly. More specific terms were assigned lower score values of PC1.

**Table 1:** Correlation of principal components

*Fraction*	*F1*	*F2*	*F3*	*F4*
Cytosolic	34,82%	34,57%	30,53%	0,06%
Membrane	35,12%	60,66%	3,93%	0,28%
Nucleus	29,21%	3,83%	63,11%	3,83%
Non-specific	0,83%	0,92%	2,41%	95,82%

Groups of accumulated points in the PCA bi-plot have similar *R *scores with respect to certain PC variables. The specificity level, defined here as the number of predecessors of the GO-term, is therefore related to the position (PC coordinate) of the group within the bi-plot in relation to that principal component.

Taken together:

(1) The specificity level of a certain GO-term is coded in the *R *score.

(2) The score-plot allows identification of GO-terms, which are characteristic for a PC. These represent PC-correlated classifications of experimental data, in our case cytosolic-, membrane- and nucleus cell fractions.

(3) The measure of specificity of terms within the dataset can be detected by PCA on the *R*-score.

### Interpretation of PCA plots obtained for different cellular fractions

The bi-plot delineated several groups, which were clearly identified by their position in the plot (Fig. 1). The numbers of identified GO-terms, which belonged to certain groups, were added together. The bi-plot of the first versus second and third PC, respectively, resolved the GO terms by specificity level via PC1. Nuclear, cytosolic, and membrane groups were identified by their loading values, marked red in the plot (Fig. 1). As expected, we found "clean" groups, which unequivocally represented the nuclear-, membrane- and cytosolic-fraction. In addition to these three groups, we detected a group consisting of a mixture of fractions. This group represented terms, which were not associated with specific sub-cellular fractions. Interestingly, many terms, which initially appeared in the background fraction (as described in section 2.1), were resolved by PCA. Thereby mixed fraction groups such as *nucleus and cytosolic *were formed and listed in table 2. The overlap of terms between "clean" and mixed groups was negligible. As expected, PCA revealed 12 different groups (number of possible permutations of the four different fractions grouped in pairs). Table 2 also showed that the majority of GO terms accumulated in mixed groups. Furthermore, each "clean" or mixed accumulation group was resolved by values of the PC loadings. Terms with a lower PC 1 score were less general than terms with higher scores. Within all groups, terms with a high *R *score represented the most unspecific GO terms, i.e. those with the least number of preceding nodes.

**Table 2:** Number of GO-terms resolved by PCA and identified within certain fractions

*Fractions*	*PC1 vs PC2*	*PC2 vs PC3*
Cytosolic	12	12
Membrane	28	28
Nucleus	3	3
Non-specific	144	221

Cytosolic-Non-specific	32	32
Membrane-Non-specific	43	81
Nucleus-Non-specific	15	15

Nucleus and Cytosolic	11	4
Membrane and Nucleus		2
Cytosol and Membrane	2	

### Comparison of PCA2GO with existing tools for identification of over representation

**Figure 2:**
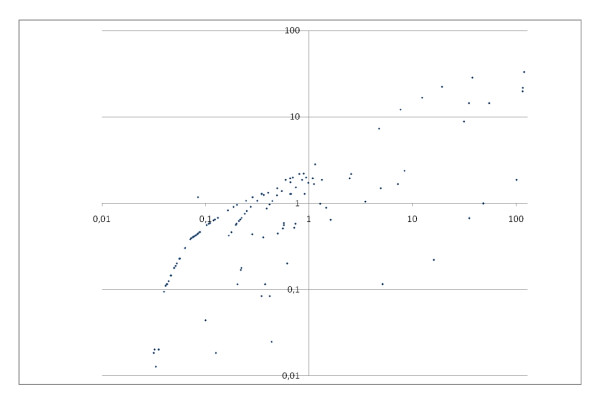
**Correlation of p-value and R-score**. This plot shows *log*_10_*p*-values on Y axis versus PCA2GO score on X axis for cytosolic fraction. PCA2GO score was scaled to interval [0-100]. Correlation coefficient was determined as 0.66.

**Figure 3:**
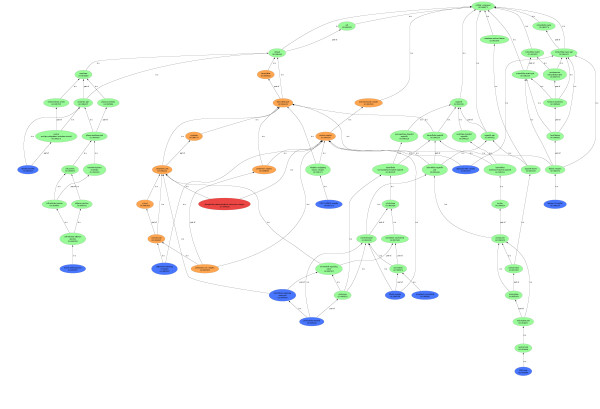
**Graphical output of GOrilla for the cytosolic fraction**. This GO-graph generated by GOrilla was generated to compare it to PCA2GO. We tested the cytosolic specific proteins against the background of all 1836 proteins.

**Figure 4:**
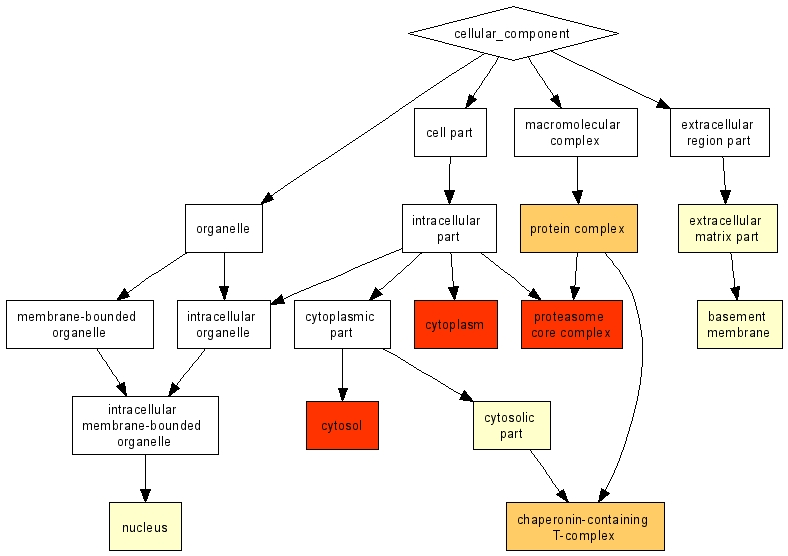
**Comparison of enriched GO terms by BINGO and PCA2GO**. This figure shows a comparison of the cytosolic fraction of the enriched BINGO (orange) and the more specific PCA2GO terms (blue). One term was found to be enriched and specific by BINGO and PCA2GO (red).

Although a direct comparison of the *R *score, which provides a weighted frequency of a GO-term in a protein set (in this case, a fraction) with *p*-values (reflecting the probability of finding the frequency of a GO-term in a protein set just by chance) is inappropriate, because different quantities are measured, we wanted to demonstrate what our findings by PCA and *R *score have in common with results obtained by standard GO analysis and what differentiates them from them. For this purpose *p*-value based enrichment analysis tools like BINGO http://www.psb.ugent.be/cbd/papers/BiNGO/ and GOrilla http://cbl-gorilla.cs.technion.ac.il/ were used. We performed a *p*-value based enrichment analysis using the protein lists from each of the "uncontaminated" fractions. The "clean" cytosolic fraction list yielded 211 proteins, the "clean" membrane fraction list 380, and the "clean" nucleus fraction list 52. The *p*-values were calculated for each exclusive protein list individually. These lists did not include proteins from common GO terms. Figure 2 shows a plot of the loged10 BINGO *p*-values versus our *R *score. The *R *scores were pre-scaled by a factor of 100 in order to obtain comparable orders of magnitude for *R*-scores and *p*-values. We found a correlation between our *R *score and the BINGO *p*-value for over representation. The correlation coefficients for the cytosolic and the membrane fraction were 0.66 and 0.64, respectively. The corresponding nucleus fraction was not analyzed because the *p*-value based over represented term list contained only one term. Since a PC analysis divides all annotated terms into observation dependent groups, we compared the specific groups, as identified by PCA, with significantly overrepresented GO terms from the BINGO analysis. BINGO detected 11 significant over expressed GO terms for the cytosolic fraction, 48 significant over expressed GO terms for the membrane fraction, and one significant over expressed GO term for the nucleus fraction. Similar results were obtained by GOrilla. Since GOrilla, in contrast to BINGO, also provided a clear visualization of the over represented terms http://cbl-gorilla.cs.technion.ac.il/, we used the graphical output of the Blast2GO tool [9] to visualize the terms detected by BINGO as shown in figure 3. The genuine output from GOrilla cytosolic fraction [8] is shown in figure 4. PCA group terms sorted by *R *score unraveled the most highly represented terms within these groups due to the correlation of the *p*-values with our *R *score. Furthermore, we compared direct enrichment lists of cytosolic fraction obtained by BINGO and GOrilla to over represented GO terms detected by PCA2GO to highlight the differences in specificity between our method and commonly used GO enrichment analysis tools. In figure 4 the combined graph represents the terms enriched using BINGO (marked orange) and PCA2GO (terms marked in blue). The graphical output was generated with the Blast2GO-tool[9]. Only one term detected by PCA2GO and BINGO is found and marked red. The figure 3 shows the resulting graphical output of an enrichment analysis by GOrilla. In comparison, a close examination of figure 4 reveals that PCA2GO, in contrast to the two other methods, marks GO terms of a higher specificity level, child terms further down the graph while both *p-*value-based calculations find statistically significant over representation only for their predecessor terms. PCA2GO evaluates the multidimensional dependencies and their mutual interactions of cytosolic fraction GO terms with other fraction terms whereas the *p*-value analysis is only based on a univariate comparison of gene enrichment in a certain GO graph normalized with a user-defined background. Only two terms (*chaperonin-containing T-complex*, *phosphoribosylaminoimidazole carboxylase complex*) are detected by BINGO and GOrilla enrichment analysis. Both terms are positioned at level 6 in relation to the root term. These terms are found to be very specific for the cytosolic fraction and also highly enriched. As shown in figure 4 the majority of relevant terms found by PCA2GO are child-terms of more unspecific-terms detected by BINGO and GOrilla. A similar effect is seen for GO terms grouped in table 3. These relations become even clearer upon analysis of the GO term cytoplasm enriched by BINGO and GOrilla. One can use our method to identify an indirect connection of cytoplasm with other highly enriched GO terms using the full structure of the cytosolic GO-DAG (shown in figure 4) and specifically inspecting the child-terms of cytoplasm. These are *chaperonin-containing T-complex (GO:0005832)*, *microtubule organizing center part (GO:0044450), pericentriolar material (GO:0000242), phosphoribosylaminoimidazole carboxylase complex (GO:0009320)*. Taken together our comparison clearly indicates that PCA2GO is on one hand a versatile tool to simultaneously uncover specific GO-terms for interpretation of experimental data and on the other hand is able to identify "over -represented" proteins via GO-branch. In this context, the over representation is not based on a statistical probability like the *p *values, but the resulting fractional groups consisting of GO term lists are correlating to one another as revealed by PCA group formation.

**Table 3:** Overview of the GO-resolving capabilities of PCA2GO and other tools

Identified to be enriched by	GO-Terms	Connection with terms detected by PCA2GO
BINGO	*intracellular, intracellular part*	*chaperonin-containing T-complex, microtubule organizing center part, pericentriolar material, UBC13-MMS2 complex phosphoribosylaminoimidazole carboxylase complex*
BINGO, GOrilla	*macromolecular complex, protein complex*	*UBC13-MMS2 complex, posphoribosylaminoimidazole carboxylase complex, angiogenin-PRI complex, charperonincontaining T-complex*
BINGO, GOrilla	*cytoplasm, cytoplasmic part*	*phosphoribosylaminoimidazole carboxylase complex, chaperonin-containing T-complex, microtubule organizing center part, pericentriolar material*
BINGO, GOrilla	*cytosol, cytosolic part*	*charperonin-containing T-complex*
BINGO, GOrilla	*Extracellular matrix part, basement membrane*	*laminin-10 complex*

## Discussion

The *R *score in combination with PCA allows comprehensive identification of correlations between different datasets. Therefore, a comparison of different datasets regardless of inherent complexity is possible. Virtually no limitations exist for the dataset and the experimental variable, which one might choose to measure representation in a GO term. Our score measures the specificity of GO terms in an experimental dataset. PCA2GO does not extend the list of over represented GO terms obtained by standard *p*-value based enrichment analyses, but detects the most specific terms, which are unique for a group. Proteins related to these specific terms can be directly identified. Identification of specific terms will also facilitate detection of over-representation at a higher tree level. To our knowledge no other GO analysis tool generates this information. Our method is especially suited for complex experimental settings due to the multivariate character of PCA. All samples are processed simultaneously and ordered, whilst taking into account the explained variance of the Principal Components. Thereby, relevant groups of GO terms are identified. In this case, specific groups of GO terms for each cell fraction, as well as mixed groups are identified. Clearly, such information assists biological interpretation of data and allows direct comparison of proteins related to these groups of GO terms, which makes a comparison between different experimental settings possible. Other GO analysis methods do not allow these direct comparisons since each sample has to be analyzed separately yielding lists of GO terms, which are only comparable to one another if the background-data (zero reference) for the samples is identical. In our method the score *R *already includes an uniform background (the total GOA). Therefore, the principal component analysis needs no specific background data.

## Conclusions

PCA2GO is a sensitive and powerful tool to detect over representation of specific GO terms in a complex DAG, which cause GO enrichment at a higher level. The identification of these terms is due to the multivariate property of PCA. The score R sorts the GO terms by statistical weight associated to the root ontology, to which the GO term belongs. The Score *R *combines the relative frequency of assigned GO-term *I *related to protein *x_i _*with a normalisation against all GO annotated proteins (see methods section). In combination with PCA an analysis of GO terms can be performed and lists of over represented GO terms are given, emphasizing the most specific terms, which are unique for a group of proteins, thereby facilitating biological interpretation of experimental data. PCA2GO works complementary to known tools like GOrilla or Bingo and delivers additional information about what causes GO-enrichment e.g. biological processes or cellular component, etc. This cannot be done by *p*-value analysis alone. The advantage of PCA2GO is the detection of highly specific, pre-enriched GO terms, which are not significantly represented in their GO level, but may lead to an over representation of this GO branch on a higher level. These terms cannot be detected by standard statistical based tools. Other pre-enriched terms will trigger enrichment in GO graph on a higher level, where hundreds of proteins appear as a kind of noise.

In summary we conclude that:

- PCA2GO is very sensitive to detect GO terms using a small number of annotated proteins.

- GO terms detected by PCA2GO are usually connected indirectly to less specific terms found to be enriched by other statistical based methods.

- Small numbered terms containing few proteins facilitate backtracking from GO terms to proteins and identification of important proteins, which might not be discovered at a lower GO-tree level.

- PCA2GO has a high *resolution power *and detects experiment specific GO terms.

Our method can be seen as a complementary tool to existing GO enrichment analysis tools featuring the detection of experiment specific GO terms. In conjunction with GOrilla or BINGO, our tool provides an enhanced *resolution *to detect specific GO terms. It is predestinated for use in high throughput experimental settings and derived high throughput data and hence represents a significant improvement compared to previous approaches.

## Methods

### Generation of dataset

Proteins for enhanced liquid chromatography-mass spectrometry (GeLC) analysis were derived from mouse cardiomyocytes. Cardiomyocytes were isolated by collagenase perfusion of intact hearts and subjected to subcellular fractionating resulting in a cytosolic, membrane and nuclear fraction (ProteoExtract^® ^Subcellular Proteome Extraction Kit, *Calbiochem*). Protein extracts were processed and mass spectrometry was performed as described [2]. Briefly, all LC-MS/MS measurements were performed with an LTQ-Orbitrap Hybrid (Thermo Fisher Scientific) combined with an Agilent 1200 nanoflow HPLC system. The mass spectrometer was operated in the data-dependent mode to automatically measure full MS scans and MS/MS spectra. Peptides were identified by searching against the International Protein Index sequence database (mouse IPI, version 3.24) using the Mascot search algorithm http://www.matrixscience.com. Mass spectra were analyzed by the MaxQuant software package [11,12], which performs peak lists and false positive rate determination [13]. To analyze the distribution of proteins in each fraction relative to the total number of proteins all detected peptides per fraction were combined. To obtain reliable results for each subcellular fraction, proteins were only sorted into specific fractions when they showed 30% enrichment in the nucleus to cytosolic- or membrane to nucleus ratio and 50% enrichment for the membrane to cytosolic ratio. Remaining proteins, which displayed no clear enrichment in one specific cell fraction, were grouped as non-specific proteins. GO terms were allocated to proteins in each fraction to analyze the distribution of allocated gene products per GO-term. For GO annotation the standard files for mouse as provided by Gene Ontology were used http://www.geneontology.org/GO.current.annotations.shtml.

### Definition of R score and enrichment of GO-terms

Our topology score *R_i _*is defined for each *i*-th GO-term. The score considers the level of the GO-Term in the graph, being a subset of the complete GO-DAG and representing the experimental data. This means R is a measure of how general or special one term is in relation to the root term.(1)(2)

With *N_exp _*being the total number of detected proteins in the experiment and *M_GOA _*representing the total number of proteins annotated by the Gene Ontology Annotation (GOA). For this purpose the source files were used to generate the whole GO structure to provide the right values for *y_i _*and *M_GOA_*. The number of *N_exp _*is important to decide, whether scaling is required or not. The exponent 2 expands the distribution of GO annotated proteins. Both variables *x_i _*and *y_i _*represent the total number of proteins. Specifically *x_i _*is the number of assigned proteins in the experimental dataset to a specific term *i*, taking all proteins assigned further down the DAG into account. *y_i _*represents the number of all possible proteins, which are assigned to the *i*-th GO term, also counting successor protein GO annotations. Multiple assigned proteins are counted only once. The quotient  is the relative frequency of assigned proteins *x_i _*in a term with the total number of N_exp _of proteins in the dataset. The second factor in the definition of r, defined as , serves as a kind of normalization against all GO annotated proteins and is the reciprocal value of the relative frequency of the *i*-th GO term referred to the total number of all GO annotated proteins. The weighting of the two factors by taking the square root of the second and quadrature of the first factor is done because the annotated proteins representing the experiment constitute only a small subset of the complete GO annotation.

### Pre-PCA transforming and Post PCA Group extraction

The localization of specific groups and the extraction of GO-terms after PCA was enhanced by global scaling: First, the score *R *was transformed by log_2 _to allow an almost linear distribution of *R-*values for all fraction datasets prior to PCA analysis. Terms, which were not represented in a certain dataset were assigned a default finite *not a number*-value after log-transformation before performing the PCA. These values can be used for *scaling*, resulting in an enhancement of the measure of correlation within the dataset. Setting all *not a number*-values to a certain finite value avoids the log-zero-problem. The *not a number *value was varied iteratively from a starting value of -10 until a clear separation of groups was achieved in the *bi-plot*.

### Introduction to PCA

The idea is to map the investigated complex system from a multidimensional space to a reduced space spanned by a few principal components (PCs) thereby revealing the principal and most important features, which underlie the data set. Consider a GO set with *p *GO-terms and let *x *= (*x*_1 _*x*_2 _... *x_p_*)' be a *p *× 1 vector, where *x_i _*is a variable for GO representation values of the *i*-th GO-term and *t *denotes transpose of a vector. Let Σ be the covariance matrix of *x *with dimension *p *× *p*. The eigenvectors and eigenvalues of Σ are defined as vectors *α_l _*and scalars λ*_i _*such that Σ*α_i _*= *λ_i_α_i_*, *i = *1,*..., p*. The first PC score (PC1) is a scalar defined as the linear function *α^t ^*= *α*_11_*x*_1_+*α*_12_*x*_2_+⋯+*α*_1*p*_*x_p _*of elements of *x *having the maximum variance among all linear functions of ***x ***(Jolliffe, 2002). Without loss of generality, assuming *λ*_1_≥ *λ*_2_≥ ⋯ ≥ *λp*, then it can be shown, that the vector of coefficients *α*_1 _for the first PC score is the eigenvector corresponding to largest eigenvalue of Σ and variance Σ*α^t ^*= *λ_p_*. The set of coefficients {*α*_11 _,..., *α*_1*p*_} are called the loadings of the first PC. An estimated value for the coefficients{*α_i, j _*= 1 ,..., *p*} (eigenvectors) of the PC scores on a set of GO-terms can be computed using singular value decomposition (SVD) (Jolliffe, 2002). Briefly, let *X *be a *N *× *p *matrix with columns corresponding to standardized GO representation values (with mean 0 and variance 1) of a group of GO-terms. The *k*-th PC score is *z *= *Xα*^*k*^, where *α_k_*, is unit length eigenvector of the covariance matrix *S*= *X^t^X*/(*N*-1) corresponding to the *k*-th largest eigenvalue λ*_k _*and *var *Σ = λ*_k _*Furthermore let *r *= rank(*X*).

Then using SVD *X *can be represented by(3)

where *U *=_u_1_, u_2_,..., u*_r _*_ is an *N *× *r *matrix, where u*_k _*= *l*^-1/2*k*^*Xαk *is scaled *k*-th PC score. These are linear combinations of GO values corresponding to columns of matrix X.  is an *r *× *r *diagonal matrix where *l_k _*is *k*-th eigenvalue of *XtX*, *A*= {*α*_1_, *α*_2 _,..., *α_r_*}is a *p *× *r *matrix where *α_k _*is eigenvector of covariance matrix S, which are also coefficients defining PC scores. Note that since the *k*-th eigenvalue of the covariance matrix *S *is *λ_k _*= *l_k_/*(*N*-1), we have var(u*_k _*) = 1/(*N*-1). Therefore, SVD provides not only the coefficients and Single Decompositions (eigenvalues) for the PCs through *A *and *L*, but also the PC scores of each observation by matrix *UL*. For simple models, it can be shown that the PCs provide an optimal approximation to the original variables (Jolliffe, 2002). Graphs plotting the *i*-th Principal Component versus the *j*-th component are usually called score-plots, which result in bi-plots if the *Loadings *are included. Bi-plots reveal the correlation of PC scores with the loading-variables. Correlated scores form groups of PC scores, which are usually clearly discernible in both types of plots.

## Authors' contributions

MB wrote the perl and R scripts, designed and developed the workflow of the method. MB and ML developed the theoretical background of the method. FC and JH helped improving the method and writing the manuscript. AK worked with the cardiomyocytes and prepared the samples for mass spectrometry. AK and MK provided the data from mass spectrometry and did the whole mass spectrometry measurements. TB, ML, FC and MB wrote and approved the manuscript. All authors read and approved the final manuscript.
